# Death of Dr. Joshua R. Lothrop—Resolutions of the Medical Staff of the Buffalo General Hospital

**Published:** 1869-08

**Authors:** J. N. Brown

**Affiliations:** Secretary


### Editorial Department.

### Death of Dr. Joshua R. Lothrop—Resolutions of the Medical Staff of the Buffalo General Hospital.

At the last regular meeting of the Medical Staff of the Buffalo General Hospital, the following resolutions were unanimously passed:

Whereas death has recently removed one of the members of the Medical Staff of the Buffalo General Hospital, Dr Joshua Rich Lothrop, who died July 22d, 1869,

*Resolved*, That in the death of Dr. J. R. Lothrop, we have to mourn the loss of a warm-hearted, unassuming, generous friend and associate, whose life and character was such as to command our highest respect, and whose early and sudden death impresses us with the deepest sadness and sorrow.

*Resolved*, That the Buffalo General Hospital thus sustains the loss of a faithful, scientific and experienced surgeon—a man of calm deliberation, sound judgment, and great skill, whose valuable services have largely contributed to the honor, success and usefulness of the institution.

*Resolved*, That we deeply sympathize with the family of our late friend, and hereby extend assurance that we shall long cherish with them his memory, associated as he is with our pleasantest professional recollections.

*Resolved*, That these resolutions be published in the Buffalo Medical Journal and transmitted to the family of the deceased.

J. N. Brown, Secretary.

